# Finite element analysis of the mechanical behavior of 3D printed orthodontic attachments used in aligner treatment

**DOI:** 10.1038/s41598-024-65864-x

**Published:** 2024-06-27

**Authors:** Riham Nagib, Andrei Zoltan Farkas, Camelia Szuhanek

**Affiliations:** 1 Department of Orthodontics, Orthodontics Research Center ‘ORTHO CENTER’, “Victor Babeş” University of Medicine and Pharmacy Timişoara, Eftimie Murgu Sq. 2, 300041 Timişoara, Romania; 2 Department of Mechatronics, University Politehnica Timişoara, 1 Mihai Viteazu Ave, 300222 Timişoara, Romania

**Keywords:** Clear aligner, Orthodontic attachments, 3D printing, FEM, Medical research, Materials science

## Abstract

The composite attachment loss during orthodontic clear aligner therapy is an adverse event that commonly happens in clinical practice and can affect the overall outcome and length of treatment. The aim of our research is to provide a basis for the further study of an innovative digital protocol and application method for orthodontic aligner attachments. Two 3D models were designed, one based on the proposed protocol and the other on the conventional method for aligner attachment application. Four attachment shapes were used to identify the maximum values for the von Mises equivalent stresses, the maximum displacements values and the areas in which these values were recorded through FEM analysis. The results of the mechanical simulation show lower values of von Mises stress recorded in the 3D printed attachments assemblies, independent of their shape, when simulated under the same boundary and load conditions. The trapezoidal prism shaped 3D printed model has a 3.7 times smaller displacement value (0.088 [mm]) compared to the adhesive resin model (0.326 [mm]). In conclusion, the proposed protocol for aligner attachments and the introduction of innovative materials is a promising method of solving conventional attachment problems in current orthodontic treatments.

## Introduction

In modern orthodontics, due to an increased demand for aesthetics and health, orthodontic clear aligner treatments have gained popularity among adult and adolescent patients^1^. This type of treatment requires digitally designed attachments, made of polymeric resins^2–4^, applied on the enamel surface that maximize the contacts between the trays and the teeth, providing the orthodontic force and improving the retention of the aligner^5,6^.

The research directions in the field, at present, are focused on determining the forms and dimensions of clinically effective attachments^7–10^, the development of new materials^11–13^ and application methods^14,15^, reducing attachment loss during treatment^16,17^ and increased stability^18,19^.

Accurate application of attachments in the treatment with braces and their resistance is crucial to obtain the desired tooth movement^15^. The same principles apply to orthodontic aligner attachments, although conventional bonding procedures which use thermoformed templates, lack precision and often create resin overflows, producing excess on the enamel surface^20^. Therefore, after the debonding of brackets the volume and surface of remnant adhesive is smaller compared to the removal of multiple composite aligner attachments in the final stages of contemporary aligner treatment^21^. It is also important that this transfer process results in a precise reproduction of the digital attachment's shape and dimension to ensure a proper fit of aligners^14^.

The aim of our research is to simulate and test the mechanical behavior and proprieties of orthodontic aligner attachments obtained using an innovative protocol, comprising of CAD design, 3D printing and adhesive application, thus broadening the scope of biomaterials used in the production of these attachments and improving the shortcomings of conventional protocols used in current aligner treatments.

## Materials and methods

### Proposed protocol for 3D printed orthodontic aligner attachments

In the proposed protocol the aligner attachments were bonded to the tooth surface by a 200 [μm] thick layer of adhesive flowable resin, as opposed to the conventional technique in which the whole attachment would be constructed out of the afore mentioned flowable resin. Models of a central upper incisor, four attachments of various commonly used shapes (semi-ellipsoid, semi-sphere, cuboid, trapezoidal prism) and the adhesive resin between them were 3D modeled.

**Figure 1:**
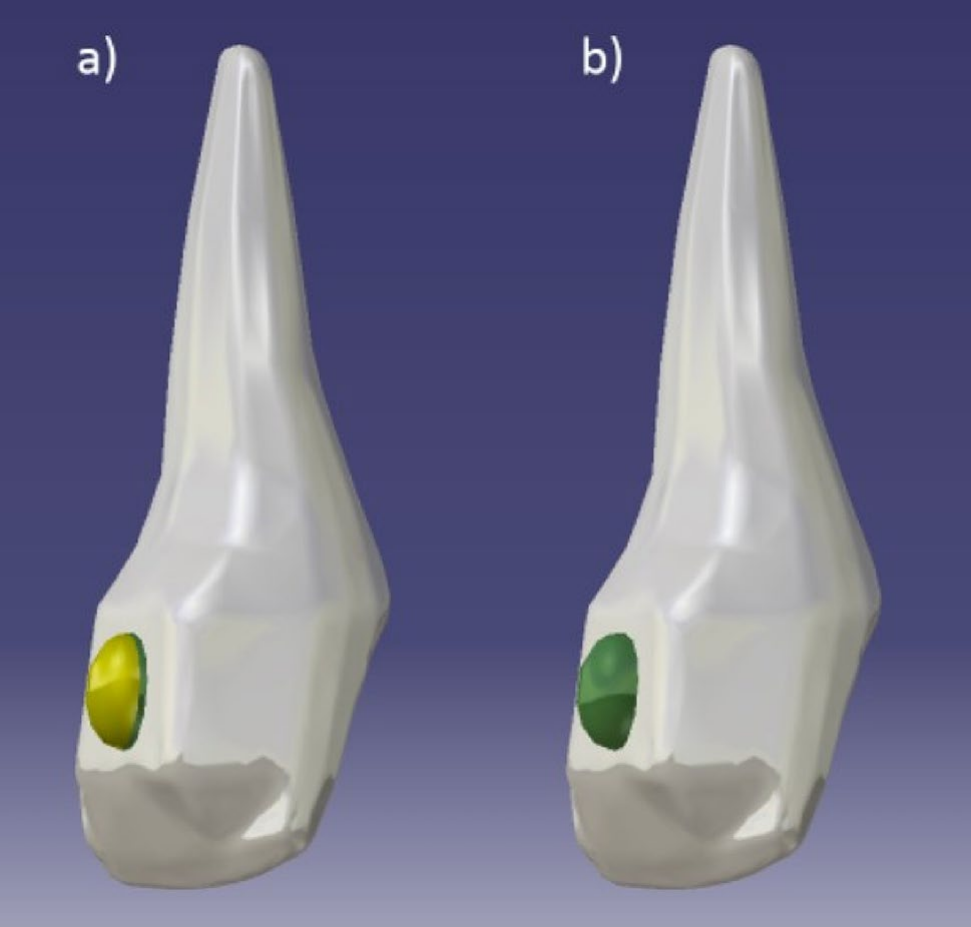
3D model of the upper central incisor and an semi-ellipsoid attachment: (**a**) 3D printed attachment—flowable adhesive resin model; (**b**) attachment fully constructed out of flowable adhesive resin.

The 3D models were assembled to reproduce the vestibular attachments used in aligner orthodontic treatment for both protocols: 3D printed attachment bonded with adhesive on the tooth surface and attachment fully consisting of adhesive composite resin (Fig. 1).

**Figure 2:**
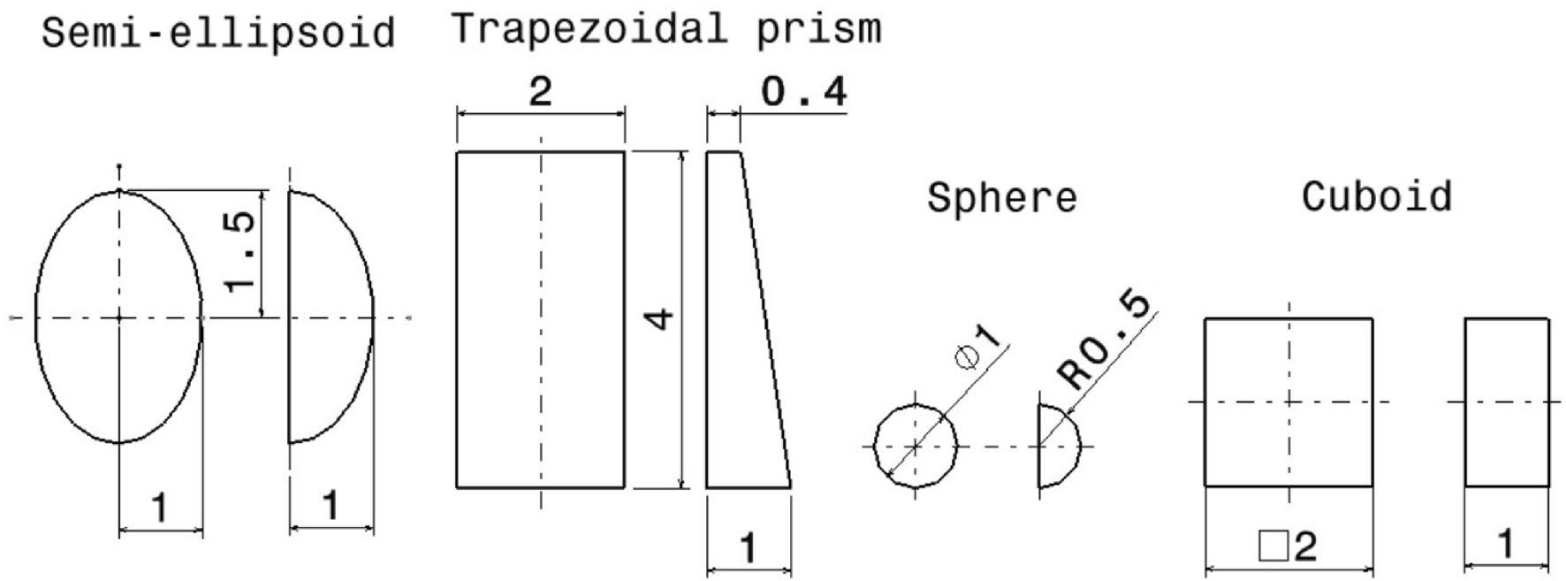
Shapes and geometrical dimensions (length unit = [mm]) of the modeled aligner attachments used in the study.

The geometrical dimensions of the shapes used were: semi-ellipsoid (major radius 1.5 [mm] and minor radius 1 [mm]); semi-sphere (radius 1 [mm]); trapezoidal prism (2 mm by 4 mm base with a 400 [μm] extrusion at the top and a 1 [mm] extrusion at the bottom); cuboid shape (2 [mm] sided square extruded 1 [mm]) (Fig. 2).

### Finite element analysis of proposed 3D printed versus conventional attachments

Using Abaqus FEA (Dassault Systems, France) analysis software, a series of simplified incisor-adhesive-attachment assemblies were modeled and used in the FEM simulations. The adhesive layer between the enamel and the dental attachments has a thickness of 200 [μm]^22^ that extends over the entire contact surface between the tooth vestibular surface and the 3D printed attachment.

**Figure 3:**
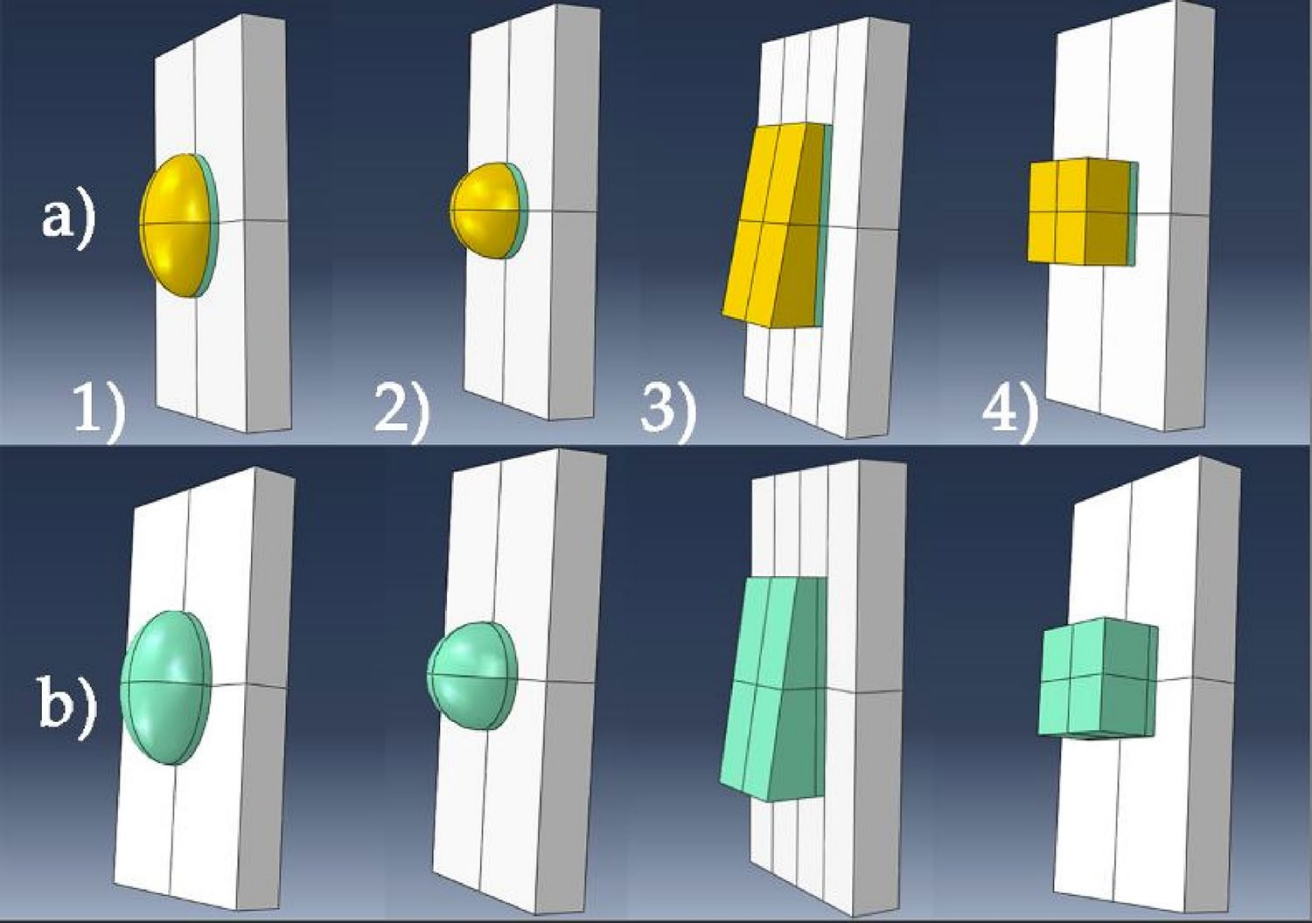
Simplified models of the four analyzed attachment shapes. (**a**) Partitions highlighted with the color yellow, represent the 3D printed attachments. The adhesive flowable resin is highlighted in green and the incisor surface is highlighted in grey; (**b**) adhesive resin (green) and incisor surface (grey). Attachment shapes for each scenario (**1**) semi-ellipsoid, (**2**) semi-sphere, (**3**) trapezoidal prism, (**4**) cuboid shape.

Instead of using the whole 3D model of the incisor, a 4 [mm] by 8 [mm] with a 1 [mm] extrusion partition was used, proper boundary conditions were applied in order to simulate the whole incisor tooth. The simplified 3D models are presented in Fig. 3.

A series of three materials were defined (Table 1). NextDent C&B Micro Filled Hybrid, which is a biocompatible Class II-a, photopolymerizable, 3D printable material developed for crowns and bridges by “Next Dent” (Vertex-Dental, Netherlands), has the following mechanical properties: an uniformly distributed density *ρ* = *1.15E − 009*, an compression modulus *E* = *2200* [MPa] and a Poisson’s ratio of *ν* = *0.3*. All values were taken from existing literature^23,24^. For the adhesive material, the mechanical properties provided by the manufacturer in the 3 M™ ESPE™ Filtek™ “Supreme XT Flowable Restorative” (Flow) technical product profile^22^, were used. These consisted of an uniformly distributed density of *ρ* = *1.84E − 009*, compression modulus *E* = *380* [MPa] and a Poisson’s ratio of *ν* = *0.3*. For the mechanical properties of the tooth model, enamel mechanical properties found in literature^8,14^ were used respectively, density *ρ* = *3.1E − 009*, compression modulus *E* = *19,613.3* [MPa], Poisson’s ratio *ν* = *0.15*.

**Table 1:** Mechanical properties of the defined materials in the simulation tests.

Material	Compression modulus [MPa]	Density [t/m^3^]	Poisson’s ratio
NextDent, vertex-dental	2200	1.15	0.3
Filtek™ “Supreme XT Flowable Restorative” 3 M ESPE™	380	1.84	0.3
Dental enamel	19613.3	3.1	0.15

Two simulations (proposed protocol and conventional protocol) were undergone for each attachment shape, using two different intensities for the applied loads in order to reproduce different exploitation conditions for the aligner attachments. This lead to a total of 16 simulations for the 4 studied attachment shapes: semi-ellipsoid; semi-sphere; trapezoidal-prism; cuboid.

**Figure 4:**
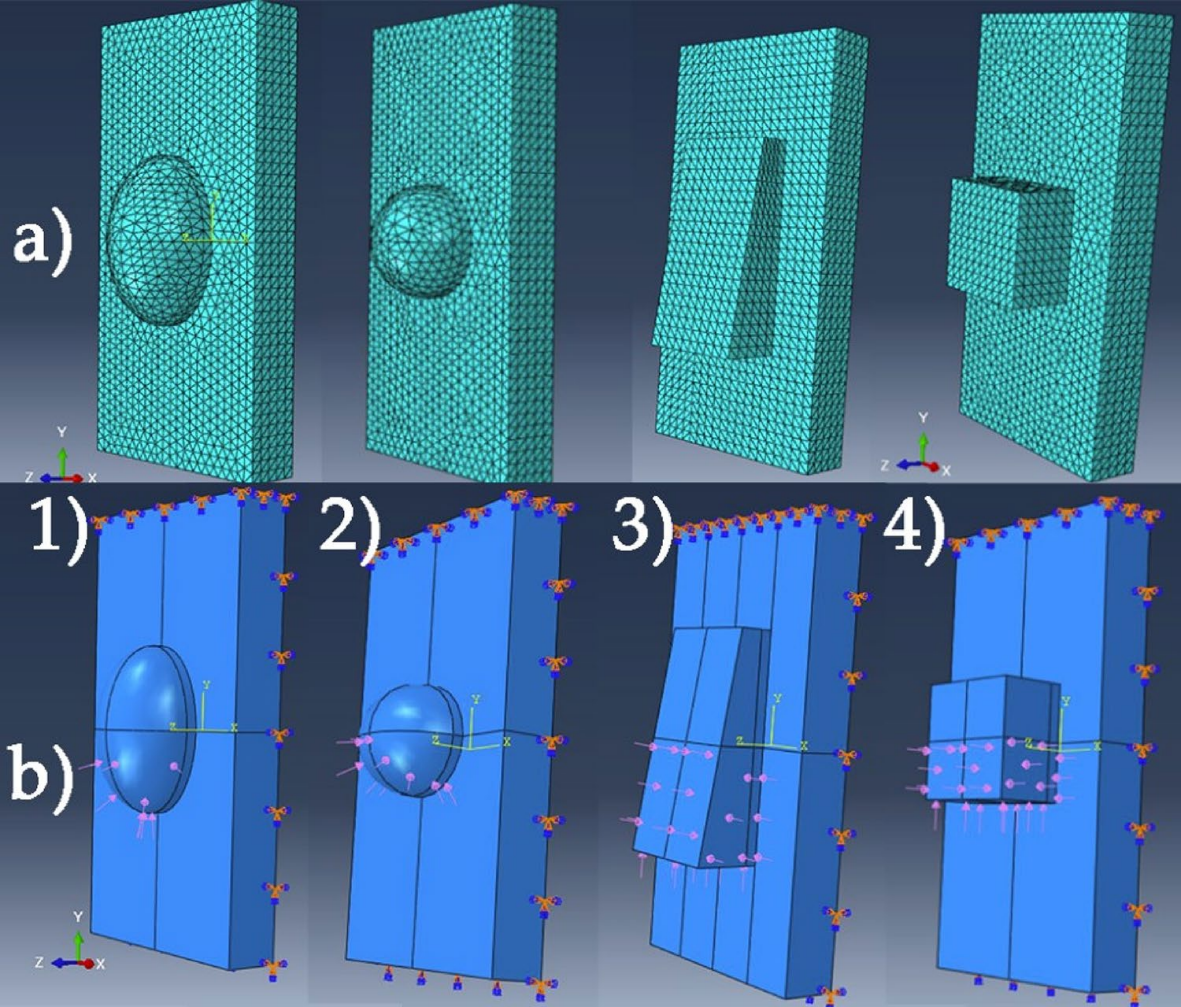
(**a**) Meshing of all four shape models: (1) semi-ellipsoid, (2) semi-sphere, (3) trapezoidal-prism, (4) cuboid; and (**b**) the applied boundary conditions and loads on each of the four shapes: (1) semi-ellipsoid, (2) semi-sphere, (3) trapezoidal-prism, (4) cuboid.

The models were meshed using an approximate global seed size of 0.2 using “Tet” shaped elements (Fig. 4a). The loads and boundary conditions are presented in Fig. 4b. On the top and posterior surfaces of the simplified tooth 3D model, an “Encastre” boundary condition was set in order to simulate the rest of the incisor body.

From the wide variety of forces that occur during aligner treatment, the chosen type of force used in the simulations was an positive uniformly distributed pressure, one round of simulations used a magnitude of 50 [N] and another round of simulations used a similar type of load, this time, with a magnitude of 10 [N]. They were applied on the lower half of the orthodontic aligner attachments used in the simulations. During aligner treatment, besides the fact that different types of forces can occur, they can be applied from different directions, if not successively from all directions throughout the orthodontic treatement. The chosen direction of the applied pressure was upwards in order to reproduce the maximum load scenario used in simulations (50 [N]).

After the material, load and constraint parameters where set, a job was created for each of the eight instances using Abaqus’s Job Manager (Dassault Systems, France).

## Results

**Figure 5:**
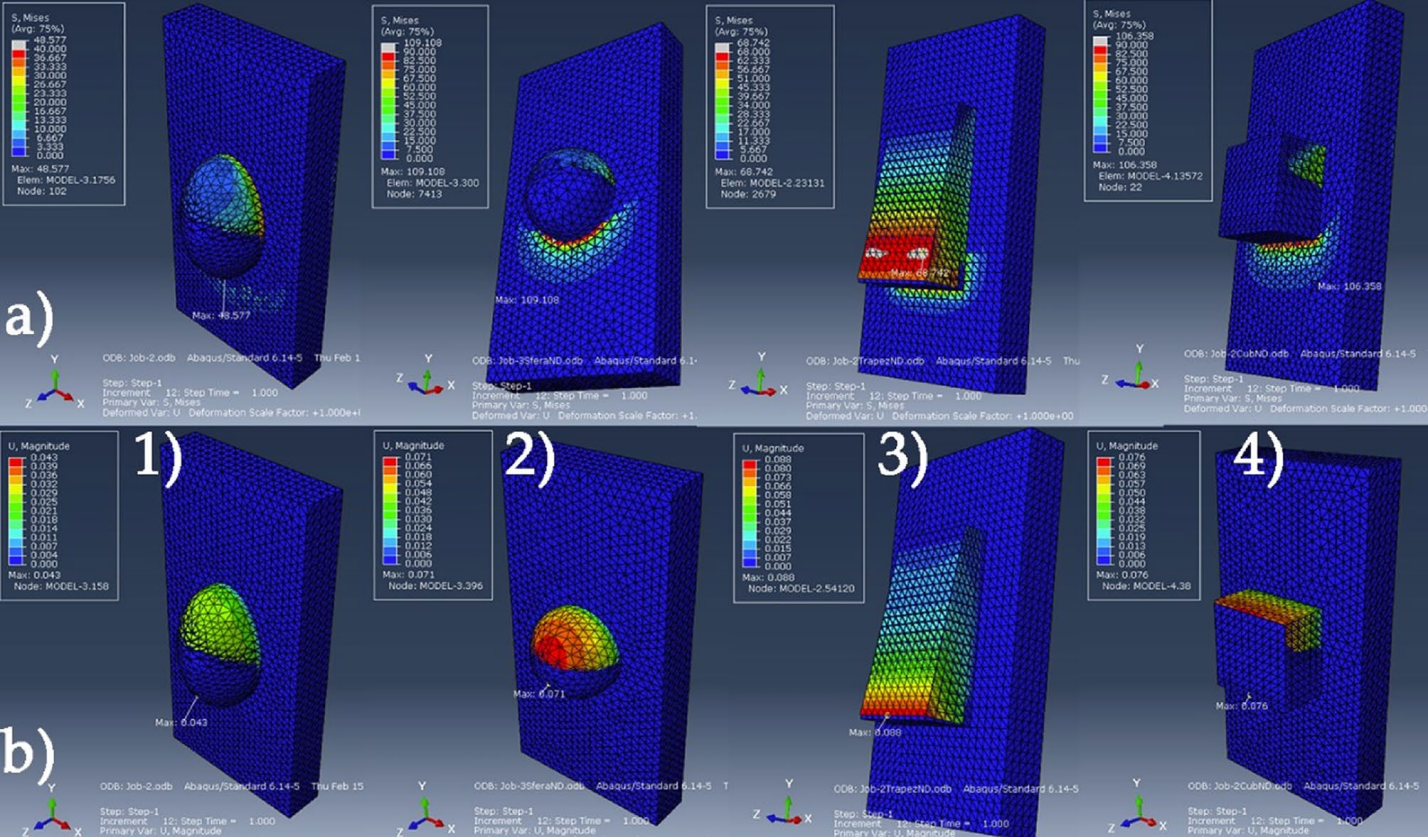
3D printed attachment-adhesive resin-enamel model: (**a**) von Mises stress distribution and (**b**) strain displacements for the four attachment shapes (1) semi-ellipsoid, (2) semi-sphere, (3) trapezoidal-prism, (4) cuboid.

**Figure 6:**
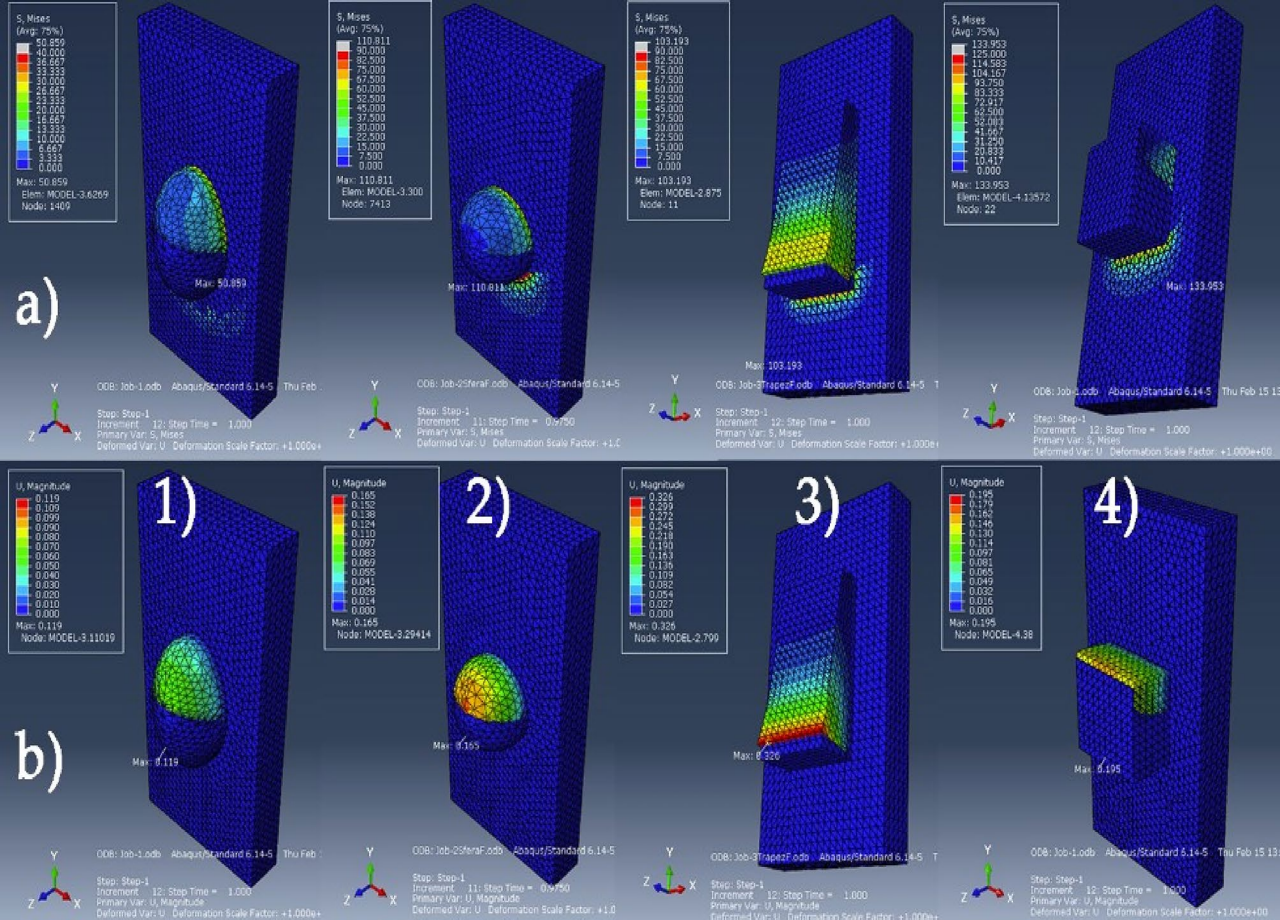
Adhesive resin-incisor model, von Mises stress distribution (**a**); strain displacements for the four attachment shapes (1)semi-ellipsoid, (2)semi-sphere, (3)trapezoidal-prism, (4)cuboid.

Simulations based on the 3D printed models, models that were assigned three different material properties: enamel—adhesive resin—3D printed resin (proposed protocol), the FEM analysis highlights the maximum values for: the von Mises equivalent stresses, the maximum displacements values and the areas in which these values were recorded in Fig. 5. FEM analysis results for the convetional protocol simulations (orthodontic aligner attachments modeled using the same material properties as the flowable adhesive resin) simulation are presented in Fig. 6.

The maximum values obtained for each attachment design, simulated using both the conventional attachment protocol and the bonded 3D printed attachment protocol are presented in Table 2.

**Table 2:** Maximum equivalent stresses and maximum displacement values for the simulated scenarios for the uniformly distributed pressure loads with a magnitude of 10 [N] and 50 [N].

	Von Mises stress NextDent + flow [Mpa]		Von Mises stress flow [Mpa]		Displacements NextDent + flow [mm]		Displacements flow [mm]	
Attachment shape	10 [N]	50 [N]	10 [N]	50 [N]	10 [N]	50 [N]	10 [N]	50 [N]
Semi-ellipsoid	9.652	48.577	10.332	50.859	0.009	0.043	0.025	0.119
Semi-sphere	22.703	109.108	25.164	110.811	0.014	0.071	0.035	0.165
Trapezoidal prism	13.821	68.742	24.149	103.193	0.018	0.088	0.068	0.326
Cuboid	21.658	106.358	29.864	133.953	0.015	0.076	0.041	0.195

**Figure 7:**
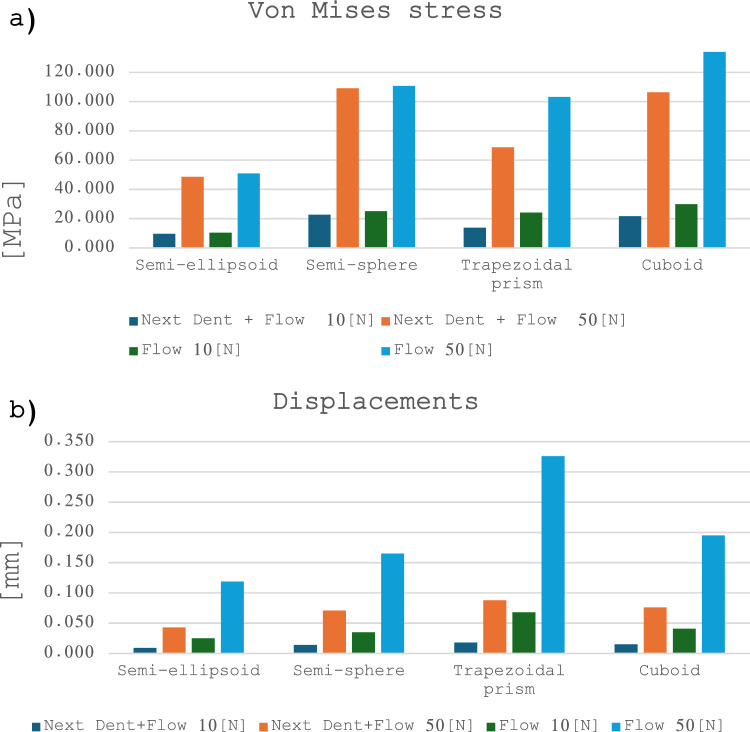
(**a**) Maximum von Mises stress distribution and (**b**) maximum displacement values for attachments made with 3D printing protocol and conventional attachments for the 10 [N] and 50 [N] loads applied.

The graphical representations of the results for the von Mises stress and the displacement values for each simulations are shown in Fig. 7.

## Discussions

Attachments are mainly used for two purposes: increasing the retention of aligners and supporting movements that occur during their application and general usage. Considering that all aligners are produced based on the attachments, which are usually bonded using initial templates, errors in initial bonding will generate unplanned tooth movements^14^. Regardless of the template thickness/material or the composite viscosity, this conventional technique presents some faults: the amount of composite necessary for attachment fabrication is not predetermined, the presence of resin overflow around the attachments^20^. Also, the precision of the attachment can only be checked after light curing and bonding on the enamel surface^13^. The proposed protocol considered in the present research addresses the precision and overflow problem by using previously 3D printed aligner attachments which are bonded to the enamel surface similar to classic fixed braces. Therefore, there would be an increase in attachment precision and a decrease in the amount of composite excess, that would require further refinement with burs and cause damage to the enamel surface.

The consistency, viscosity and other composite properties can influence the position and shape of attachments, factors that are essential for the efficacy of the attachments^5^. The 3D printable resin used to create the proposed protocol simulation models has greater mechanical proprieties than the conventional flowable composite resins used in aligner treatment.

Studies suggest that higher bonding forces lead to superior retention of the attachments^25^. The Finite Element Method numerical technique used to analyze the proposed method of 3D printed aligner attachments versus the conventional method is widely used in the orthodontic field to analyze the stress strain distribution of orthodontic appliances^5,26^ and has long been validated as a tool that provides acceptable accuracy to predict clinical outcomes^7^. Given the small contact area analized and identical simulation environments, the present study uses simplified models in FEM simulations^27^, the aim being to reduce the number of nodes and the overall output time of the analysis. The lack of a curvature between the simplified models contact surfaces is a limitation of the current study and could be adressed in future research.

Another limitation can be the fact that the simplified models used in the current research were only partitioned into separate regions on which different material properties were set, thus highlight the mechanical behavior of an ideal model without taking into consideration the adhesive properties between them. Literature sugests that 3D printed composite resin behaves similarly to other well-established definitive restoration materials^28^. Given the lack of studies on 3D printed attachments, the analisis of the adhesion between flowable and 3D printed resins represent a topic that can be explored in future research papers.

Forces used in this study were based on literature that suggested that distributed forces up to a maximum value of 10[N] cand be exerted by orthodontic aligners on attachments^29^, loads that are further distributed onto the tooth surface. Also, a value equivalent to the maximum force that could be exercited by the hand of a human operator in case of improper maneuvering^30^, during the application of the orthodontic aligner or attachment, was used in simulations. The direction of the loads was chosen to be vertical, in order to be consistent with the highest value of 50 [N], used in the current study.

The results of the mechanical simulation show lower values of von Mises stress in the case of the 3D printed attachments assemblies, independent of their shape or applied pressure, when simulated under the same boundary and load conditions. The semi-ellipsoid model made out of the 3D printed resin registered a sensibly lower von Mises stress (48.577 [MPa]) compared to the conventional attachment made out of the flowable resin (50.859 [MPa]), when the load of 50 [N] was applied. For the 10 [N] simulation, similar differences were registered. Maximum values of 9.652 [MPa] for the 3D printed model and 10.332 [MPa] for the attachment model that was assigned flowable adhesive resin mechanical properties. The semi-sphere 3D printable resin model also obtained better results (109.108 [MPa]) compared to the conventional attachment model (110.811 [MPa]) when a load of 50 [N] was applied. In the 10 [N] load simulation, a maximum of 22.703 [MPa] was registered, compared to the value of 25.164 [MPa] obtained in the flow resin model.

Higher differences between results were observed in the case of the trapezoidal prism, 68.742 [MPa] for the 3D printed material compared with 103.193 [MPa] for the conventional attachment at 50 [N], and a maximum of 13.821 [MPa] in the 10 [N] load simulation, compared to the value of 24.149 [MPa] obtained for the conventional adhesive attachment. The cuboid shaped attachment registered a maximum value of 106.358 [MPa] in the case of the 3D printable resin, compared to 133.953 [MPa] registered for the adhesive resin model at a load of 50 [N]. When subjected to a load of 10 [N], the maximum von Mises stresses were 21.658 [MPa] in the 3D printed model simulation and 29.864 [MPa] in the case of the flow adhesive model.

In orthodontics, when using bonded metallic brackets, the main failure point is the adhesive layer level^15^. While the results of the present study show the same general behavior (the location of the maximum von Mises stresses occur at the bonding layer), in the case of the 3D printed resin attachment-adhesive-enamel model, the bonded 3D printed attachment can withstand higher forces than the currently used technique for transferring aligner attachments, therefore limiting the loss of the attachments. Loss that has been reported to be an issue for both clinicians and patients alike^17,21^. Studies showing increased incidence of composite attachment loss during orthodontic clear aligner therapy, with a loss rate in molar attachments at up to 11.49%, higher than at the incisor level with almost 6%^31^.

Regarding adhesive resins losses, no significant differences in the survival rates were observed while studying the failure rate of multiple flowable composites^32^. Therefore, the proposed protocol in which we use flowable composite resin for adhesion to the enamel surface, due to the small size of the aligner attachments, should not result in greater loss rates than classic orthodontic brackets. The resistance to higher stresses of the 3D printed attachment allows the use of larger attachments, greater size offering the possibility of a continuous increase in force and momentum^33^, thus increasing the level of retention of the aligner and facilitating a better force transition in the favor of orthodontic tooth movements.

The displacement values resulted from the simulations are around 2.767 times lower in the case of the semi-ellipsoid model made out of the 3D printed resin (0.043 [mm]) than in the case of the bonding resin model (0.119 [mm]) under an applied pressure of 50 [N]. When subjected to the 10 [N]] load, the 3D printed model registered a displacement of 0.009 [mm] (2.777 times lower) while the flow model registered a 0.025 [mm]. A displacement value of 2.323 times smaller was registered in the case of the semi-sphere 3D printable resin model (0.071 [mm]) compared to the adhesive resin model (0.165 [mm]) in the 50 [N] simulation. While simulating under the load of 10 [N], the 3D model registered a displacement value of 0.014 [mm] compared to the 2.5 times bigger value of 0.035 [mm] measured for the flow model. The trapezoidal prism shaped 3D printed model recorded a 3.7 times smaller displacement value (0.088 [mm]) compared to the adhesive resin model (0.326 [mm]) in the 50 [N] simulation, while the 10 [N] simulation recorded a displacement of 0.018 [mm], compared with the 3.777 times higher displacement recorded in the flow model. The cuboid shaped models obtained deformation values of 0.076 [mm] for the 3D printed resin and 0.195 [mm] for the adhesive resin at 50 [N], an 2.5 times difference between the 2 materials. At 10 [N] they recorded a displacement of 0.015 [mm] for the 3D model and 0.041 [mm] (2.733 times difference) for the Flow model. The lower displacement values observed show that the 3D printable resin can insure the creation of a more efficient and stable orthodontic attachments.

The degree of UV curing of conventional attachments, cured under the aligner body, are significantly lower compared with attachments cured without aligners on top of them^34^. In the case of the 3D printed attachments, curing is still needed but is done after the printing process in bulk, prior clinical use, considerably reducing treatment time by needing to only cure a small film of adhesive resin (200 [μm]) instead of the whole attachment body (up to 20 s for a thickness of 1 [mm]^22^ according to literature), for each conventional aligner attachment.

## Conclusion

In conclusion, this study demonstrated quality mechanical behavior and proprieties of the aligner attachments obtained using the proposed protocol. Further development in digital protocols and the introduction of innovative materials in the process of making orthodontic clear aligner attachments, with the aim of solving current problems of the conventional procedures used in modern orthodontic treatments, represent promising horizons**.**

## Data Availability

The datasets used and/or analyzed during the current study are available from the corresponding author on reasonable request.
